# Dual-hemisphere tDCS facilitates greater improvements for healthy subjects' non-dominant hand compared to uni-hemisphere stimulation

**DOI:** 10.1186/1471-2202-9-103

**Published:** 2008-10-28

**Authors:** Bradley W Vines, Carlo Cerruti, Gottfried Schlaug

**Affiliations:** 1 Department of Neurology, Beth Israel Deaconess Medical Center and Harvard Medical School, Boston, MA 02215, USA; 2 Institute of Mental Health, Department of Psychiatry, University of British Columbia, Vancouver, BC, V6T 1Z3, Canada; 3 Harvard Graduate School of Education, Cambridge, MA 02138, USA

## Abstract

**Background:**

Transcranial direct current stimulation (tDCS) is a non-invasive technique that has been found to modulate the excitability of neurons in the brain. The polarity of the current applied to the scalp determines the effects of tDCS on the underlying tissue: anodal tDCS increases excitability, whereas cathodal tDCS decreases excitability. Research has shown that applying anodal tDCS to the non-dominant motor cortex can improve motor performance for the non-dominant hand, presumably by means of changes in synaptic plasticity between neurons. Our previous studies also suggest that applying cathodal tDCS over the dominant motor cortex can improve performance for the non-dominant hand; this effect may result from modulating inhibitory projections (interhemispheric inhibition) between the motor cortices of the two hemispheres. We hypothesized that stimultaneously applying cathodal tDCS over the dominant motor cortex and anodal tDCS over the non-dominant motor cortex would have a greater effect on finger sequence performance for the non-dominant hand, compared to stimulating only the non-dominant motor cortex. Sixteen right-handed participants underwent three stimulation conditions: 1) dual-hemisphere – with anodal tDCS over the non-dominant motor cortex, and cathodal tDCS over the dominant motor cortex, 2) uni-hemisphere – with anodal tDCS over the non-dominant motor cortex, and 3) sham tDCS. Participants performed a finger-sequencing task with the non-dominant hand before and after each stimulation. The dependent variable was the percentage of change in performance, comparing pre- and post-tDCS scores.

**Results:**

A repeated measures ANOVA yielded a significant effect of tDCS condition (*F*(2,30) = 4.468, *p *= .037). Post-hoc analyses revealed that dual-hemisphere stimulation improved performance significantly more than both uni-hemisphere (*p *= .021) and sham stimulation (*p *= .041).

**Conclusion:**

We propose that simultaneously applying cathodal tDCS over the dominant motor cortex and anodal tDCS over the non-dominant motor cortex produced an additive effect, which facilitated motor performance in the non-dominant hand. These findings are relevant to motor skill learning and to research studies of motor recovery after stroke.

## Background

Techniques that stimulate the brain non-invasively hold the promise of revealing causal relations between brain regions and brain functions [1]. Furthermore, these techniques may also facilitate skill acquisition, learning and neural plasticity [2-4]. Because it is portable, relatively inexpensive, and free from any major side-effects, Transcranial Direct Current Stimulation (tDCS) is ideally suited for use in stroke recovery therapies [5,6]. tDCS modulates regional brain activity by altering the membrane potential of neurons [7,8]. The effects of tDCS on a population of neurons are determined by the polarity of stimulation – anodal stimulation increases excitability and cathodal stimulation decreases excitability. Changes in excitability induced by tDCS are mediated by activity in sodium and calcium ion channels in the membranes of neurons, and by the efficiency of receptors for NMDA neurotransmitters [7,9].

Applying tDCS over the motor cortex has the potential to facilitate improvements in motor functioning. Research with healthy participants revealed that applying anodal tDCS over the motor cortex can improve performance for the hand contralateral to the stimulated hemisphere [10-12]. Stroke-recovery research has also explored the potential benefits of using tDCS, or tDCS in combination with physical or occupational therapy. For example, studies have reported that applying anodal tDCS to the stroke-affected motor cortex improved motor functioning; the tDCS may have stimulated preserved areas of the motor cortex to enhance synaptic efficiency along the corticospinal tract. It may also be possible to improve motor ability by applying cathodal tDCS to the motor cortex ipsilateral to the performing hand; this may have a beneficial effect in stroke patients by diminishing maladaptive inhibitory projections from the undamaged hemisphere onto the damaged motor cortex [5,6,13-15].

In our previous studies, we found that cathodal tDCS over the dominant motor cortex had a facilitative effect for the non-dominant hand [4,12]; presumably, the cathodal tDCS modulated inhibitory projections between the motor cortices of the two brain hemispheres to achieve this effect. The predominant mode of interhemispheric interaction between primary motor cortices is inhibitory [16], and there is an asymmetry in this Interhemispheric Inhibition (IHI), with stronger inhibitory projections originating in the dominant motor cortex [17-19]. Therefore, decreasing excitability in the dominant motor cortex may release the non-dominant motor cortex from inhibitory suppression, and thereby increase excitability in the non-dominant motor cortex. This could explain why applying cathodal tDCS over the motor cortex of the unaffected hemisphere facilitates motor recovery for stroke patients, particularly when the damage is to the non-dominant hemisphere [15,20].

It is possible that the ideal montage for catalyzing motor improvement may involve stimulating both motor cortices simultaneously, with an appropriate combination of anodal and cathodal tDCS. The aim of the present study was to test the hypothesis that dual-hemisphere stimulation (simultaneously applying anodal tDCS to the non-dominant motor cortex and cathodal tDCS to the dominant motor cortex) would improve finger-sequence performance for the non-dominant hand more than uni-hemisphere stimulation (applying only anodal tDCS to the non-dominant motor cortex). Results of this study are relevant to the use of non-invasive brain stimulation for facilitating motor recovery after stroke.

## Methods

### Participants

Sixteen healthy adults (mean age: 27.6, s.d.: 3.6) took part in the experiment after giving their informed written consent following protocol approved by the IRB of the Beth Israel Deaconess Medical Center (BIDMC). All participants were right handed, as determined by the Edinburgh Handedness Inventory [21] with laterality quotients ranging from 80–100, and were naïve as to the purpose of the study.

### Procedure

Participants underwent three stimulation sessions – one for each condition (dual-hemisphere, uni-hemisphere, and sham tDCS) – while sitting in an office chair. Each stimulation session was conducted on a different day, such that consecutive stimulation sessions were separated by at least 24 hours. The ordering for the stimulation conditions was counterbalanced across participants as completely as possible. Due to the number of participants (N = 16), it was not possible to divide them evenly into the six possible orderings for the stimulation conditions. Notably, the uni-hemisphere condition occurred first slightly more often than the dual-hemisphere condition.

One saline-dampened electrode (oval in shape, with area = 16.3 cm^2^) was positioned over the left motor region, centered on C3 of the 10–20 International EEG system, and another over the right motor region, centered on C4. Neuroimaging studies have confirmed the correspondence between the primary motor cortices of the left and right hemispheres and C3 and C4, respectively [22,23]; our own pilot study (N = 5) with high resolution MRI (1 mm^3 ^voxel size) provided further support. A third electrode (rectangular in shape, with area = 30 cm^2^) positioned over the left supraorbital region served as a reference electrode in the uni-hemisphere stimulation condition.

For the dual-hemisphere condition, the electrode over the right motor area was used as the anode, and the electrode over the left motor area was the cathode. For the uni-hemisphere condition, the electrode over the right motor area was the anode, and the electrode over the left supraorbital region was the cathode; this location for the cathodal electrode has been shown to be functionally ineffective in experimental designs [11]. The sham electrode montage varied randomly between that for the dual-hemisphere and uni-hemisphere conditions. Due to the size of the electrodes centered on M1 of the right and left hemispheres (16.3 cm^2^), the stimulation may have extended beyond primary motor cortex into nearby premotor and anterior superior parietal areas.

A battery-driven, constant current stimulator (Phoresor, Iomed Inc., Salt Lake City, UT) delivered the electrical current from anode to cathode. For both the dual-hemisphere and uni-hemisphere conditions, the tDCS current ramped up over the first few seconds to a maximum of 1 mA, and then remained on for the remainder of the 20-minute stimulation period. This resulted in a total current density of .07 mA/cm^2 ^over the motor cortices, and of .03 mA/cm^2 ^over the left supraorbital area. The sham control condition was identical to the dual-hemisphere and uni-hemisphere conditions, except that the experimenter reduced the current to zero after 30 seconds; the current then stayed at zero for the remaining time period. Participants reported a tingly or itchy sensation at the start of the stimulation, which typically faded away after a few seconds. This sensation was present for both real and sham tDCS. Gandiga and colleagues [24] found that naive participants were not able to distinguish between real and sham tDCS, as employed in a manner similar to the present study. Participants read a book or magazine during the stimulation periods.

### Task

Instructions for a single trial were to place the index, middle, ring, and little fingers of the left hand over the numbers two through five on a standard keyboard, and to repeat a uni-manual pattern of five sequential keystrokes as accurately and quickly as possible for 30 seconds. Subjects were shown which numbers of the numeric keypad corresponded to their fingers. During the task, the number sequence was displayed on a computer screen placed in front of the participant. The task interface did not provide any feedback about errors. The keyboard was plugged into the experiment computer by means of a USB cable. The subjects were asked to position the keyboard in a manner that was most comfortable, and to keep the same position throughout the experiment. Prior to any testing there were two warm-up trials. For testing, participants performed three trials of the uni-manual finger sequence task with their left hand before and immediately after each tDCS stimulation period. Task performance for all three trials lasted approximately two minutes including short breaks between the 30-second trials. Participants were tested with a different keystroke pattern for each stimulation condition. Within any one stimulation condition, the pre- and post-stimulation sequences were always the same. Keystroke patterns of equal difficulty were identified with pilot testing, and were randomly counterbalanced in ordering across participants and stimulation conditions. The keystroke patterns all started and ended with the same number, with the other three numbers appearing once. The following three keystroke patterns were used: left hand – 35243, 34523, 32453.

To summarize the experimental procedure and task, the participant began with a short warm-up, including two practice periods. The finger sequence used for the warm-up was not the same as any finger sequence used for experimental testing. During a stimulation session, participants performed three finger-coordination trials. Stimulation was then applied for 20 minutes, after which participants again performed three finger-coordination trials.

### Data Analyses

We calculated the dependent variable as the percentage of change, from pre-tDCS to post-tDCS, in the sum of correct sequential keystrokes over three trials. A participant could score up to four points per iteration of a sequence. For example, if the sequence were 35243, the participant scored one point for typing 3–5, two points for 3-5-2, three points for 3-5-2-4, and four points for 3-5-2-4-3.

A preliminary analysis of the entire dataset showed that there were trials across subjects and conditions that had many errors. In order to avoid skewing the analysis with these outlying data, we developed a very conservative regimen for eliminating outliers. Outliers were identified as 30-second trial periods for which the number of errors was greater than two standard deviations above the mean number of errors across all 30-second trial periods. If a trial was identified as an outlier, we not only removed that particular trial from further analyses but also the corresponding pre- or post-stimulation trial. For example, if the first 30-second period in a pre-stimulation trial set was an outlier, we also removed the first 30-second period in the corresponding post-stimulation trial set. Across all 16 participants, we removed trials for eight outlier periods in total, out of a total of 288 trials. The maximum number of outliers within an experimental condition was three, out of 96 trials per condition. The maximum number of outliers for a participant across all conditions was two, out of a total of 18 trials per participant.

Using SPSS 11, the data were entered into a one-way repeated measures ANOVA, with three levels for tDCS condition (dual-hemisphere, uni-hemisphere, sham). Planned post-hoc analyses, with two-tailed paired-samples t-tests, directly compared the effects of dual-hemisphere, uni-hemisphere and sham tDCS; though we had developed predictions based upon our previous findings [12] that would warrant using 1-tailed tests, we used two-tailed tests to adjust for the fact that each independent set of data (e.g., data for left-hand performance with anodal stimulation over the right motor cortex) was included in two post-hoc comparisons.

## Results and Discussion

**Figure 1:**
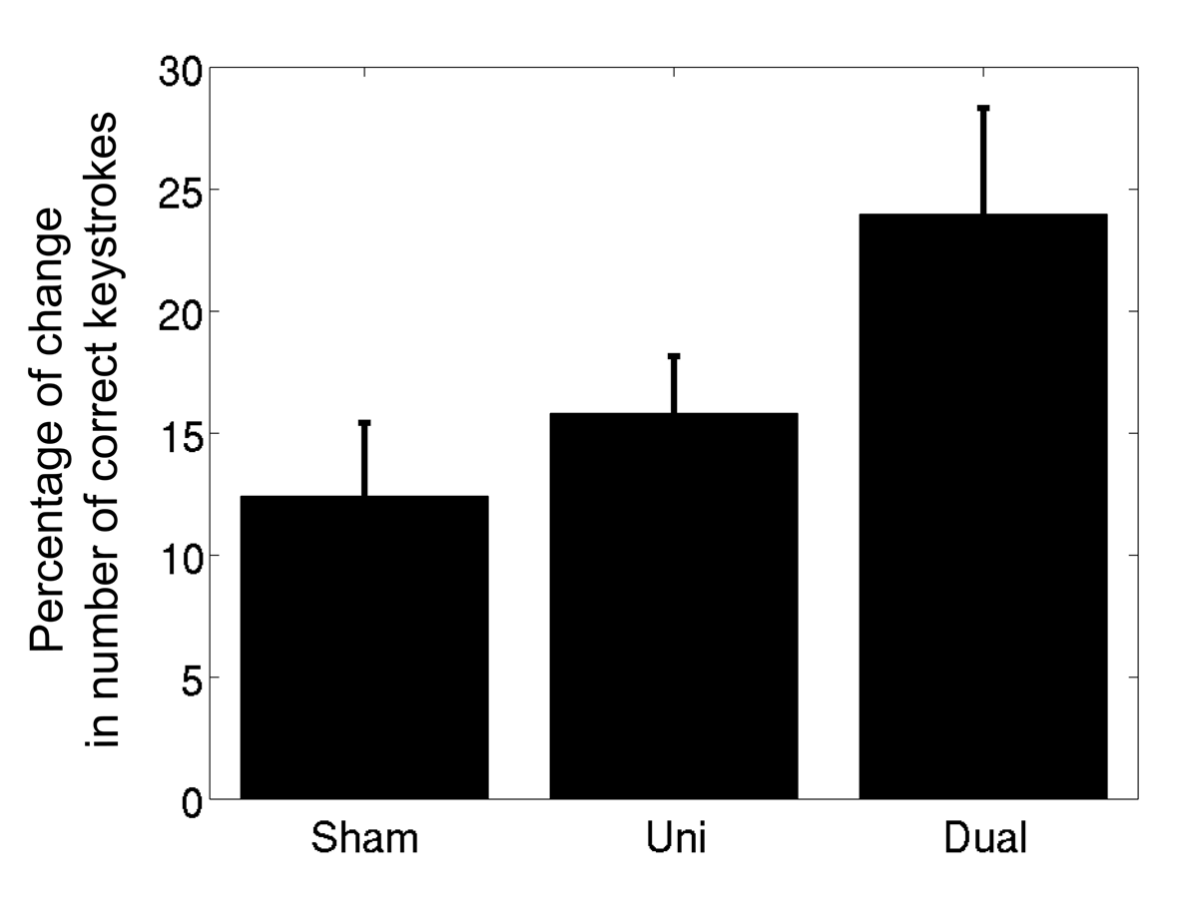
**Effects of three tDCS stimulation conditions on finger-sequence performance**. The mean percentage of change in the total number of correct sequential keystrokes across all subjects (*N *= 16) for sham, uni-hemisphere, and dual-hemisphere tDCS. Error bars signify the standard error of the mean (SEM). Dual-hemisphere tDCS improved finger-sequence coordination for the left hand significantly more than uni-hemisphere or sham stimulation.

All 16 participants completed the experimental procedures. Data for the three stimulation conditions are shown in Figure 1. The values for the dependent variable for the three stimulation conditions were the following: sham (mean = .12, SEM = .03), uni-hemisphere (mean = .16, SEM = .02), dual-hemisphere (mean = .24, s.e.m. = .04). A repeated measures ANOVA yielded a significant within-subjects effect of tDCS condition (*F*(2,14) = 4.468, *p *= .037). This result shows that the effects of tDCS depended upon the stimulation montage. Post-hoc analyses with two-tailed paired-samples *t*-tests revealed significant differences between the effects of dual-hemisphere stimulation and uni-hemisphere stimulation (t(15) = 2.58, *p *= .021), and between dual-hemisphere stimulation and sham stimulation (*t*(15) = 2.24, *p *= .041). The data for uni-hemisphere and sham tDCS were not significantly different, though there was a trend towards greater improvement with uni-hemisphere stimulation (*p *> .05). The significant effects were not due to differences in baseline scores across conditions; a one-way ANOVA with three levels (sham, uni-hemisphere, dual-hemisphere) comparing the number of points per trial at baseline yielded *F*(2,133) = .30, *p *= .75. These results show that dual-hemisphere stimulation improved motor performance for the left hand significantly more than both uni-hemisphere and sham stimulation.

Further analyses explored which aspects of finger-sequence performance contributed to significant changes in performance. Using the proportion of change in the rate of keystrokes and the absolute change in the number of errors as dependent variables, we applied one-way ANOVAs and post-hoc paired-samples t-tests (two-tailed) to compare the effects of dual-hemisphere stimulation with uni-hemisphere and sham stimulation. The ANOVA comparing the proportion of change in the rate of keystrokes across tDCS conditions (sham, uni-hemisphere, and dual-hemisphere) yielded *F*(2,14) = 4.96, *p *= .029. The improvement in performance for dual-hemisphere stimulation relative to uni-hemisphere stimulation was associated with an increased rate of keystrokes (*t*(15) = 3.133, *p *= .007), as was the improvement in performance for dual-hemisphere stimulation relative to sham tDCS (*t*(15) = 2.371, *p *= 0.032). The ANOVA comparing the absolute change in the number of errors across tDCS conditions yielded *F*(2,14) = .44, *p *= .53. Though it did not reach significance, there was a trend for decreased errors for both uni- and dual-hemisphere stimulation relative to sham. Therefore, the tDCS may have its strongest effect on the rate of keystrokes, as opposed to accuracy of keystrokes.

We found evidence that dual-hemisphere stimulation (simultaneously applying anodal tDCS over the non-dominant motor cortex and cathodal tDCS over the dominant motor cortex) improved motor performance for the non-dominant hand significantly more than uni-hemisphere stimulation (applying anodal tDCS over the non-dominant motor cortex), and also more than sham stimulation. These data support our hypothesis that dual-hemisphere stimulation facilitates motor improvement for the non-dominant hand significantly more than uni-hemisphere stimulation.

We hypothesize that IHI mediated the additional improvement in finger-sequence performance for dual-hemisphere compared to uni-hemisphere tDCS. The dual-hemisphere condition was identical to the uni-hemisphere condition, but for the addition of cathodal tDCS over the dominant motor cortex. (The direction of current over the right motor cortex may have differed slightly between uni- and dual-hemisphere stimulation, because the location of the cathodal electrode was different for the two conditions; however, Miranda and colleagues [25] found with modeling techniques that the current density on the cortical surface remains relatively homogeneous across different electrode placements.) We hypothesize that by decreasing excitability in the dominant motor cortex with cathodal tDCS, the stimulation dampened inhibitory projections from the dominant onto the non-dominant motor cortex, which released the non-dominant motor cortex and augmented the excitatory effects of anodal tDCS there. Increasing the excitability of neurons in a motor region may promote improvements in performance for the contralateral hand by facilitating long-term potentiation between activated neurons [7,26]. Practicing motor behaviors, such as finger movements, naturally heightens motor-cortical excitability [27,28]. Therefore, increasing excitability with tDCS, whether directly or indirectly, may provide a means of inducing a physiological state that supports acquiring motor skills.

It is notable that there was a trend for uni-hemisphere stimulation over the non-dominant motor cortex to improve motor performance for the non-dominant hand compared to sham, though it did not reach significance. In our previous study [12], we found an analogous trend when applying anodal, uni-hemisphere stimulation over the dominant hemisphere, and measuring performance for the dominant hand. These data, therefore, provide further evidence that contralateral effects are analogous for both hemispheres. Further research is needed to determine whether ipsilateral effects are also analogous between the two hemispheres, or if there is an asymmetry in the ipsilateral effects on motor performance [4].

We chose to focus on performance for the non-dominant hand in the present study for two primary reasons. Firstly, evidence suggests that the non-dominant hand has a greater potential to improve with practice compared to the dominant hand. Boggio and colleagues [10] found improvements in contralateral motor performance when applying anodal tDCS to the non-dominant motor area, but not to the dominant motor area. They posited that the dominant hand already performed at a ceiling level prior to stimulation, whereas the less-adept non-dominant hand had room to improve. In our previous studies [4,12], we too found larger improvements for the non-dominant hand, which suggests that the positive effects of different stimulation conditions may be most apparent in the non-dominant hand. Secondly, interhemispheric inhibition between motor cortices appears to be asymmetric, with stronger inhibitory projections originating in the dominant hemisphere [17-19]. Further evidence suggests that IHI from the dominant motor cortex has a greater impact on motor performance [29]. In light of these findings, it is unclear whether decreasing excitability in the non-dominant motor cortex would cause a corresponding increase in excitability in the dominant cortex. In future research, it would be valuable to explore asymmetries in the behavioral and physiological effects of applying tDCS over the motor areas of the two hemispheres, and to conduct a complementary study to the present study that focuses on performance for the dominant hand.

There are two important points regarding the limitations of this study. The first point is that the measures only revealed effects on behavior. We did not use methods to collect data on neural excitability directly, such as measuring motor-evoked potentials (MEP) with Transcranial Magnetic Stimulation (TMS) or by studying interhemispheric interactions using paired-pulse techniques and by testing the ipsilateral silent period [30]. Future research will be necessary to explore the neurophysiology underlying the behavioral effects found in the present study. Secondly, we did not test the effects of uni-lateral stimulation with cathodal tDCS over the dominant motor cortex. For this reason, we cannot determine how much of the improvement in the dual-hemisphere condition can be accounted for by the cathodal tDCS. This too needs to be addressed by future research.

Neuroimaging research has shown that sequential finger movements engage primary motor, pre-motor, and supplementary motor areas in the brain [31,32]. Our results reveal that stimulating a region of cortex centered on M1, in particular, can significantly influence complex motor performance. Non-invasive brain stimulation over M1 is known to affect basic motor behaviors such as speed, accuracy, and force of movement [33-35]. Post-hoc analyses in the present study revealed that the significant effects of stimulation were largely due to changes in the speed of keystrokes. Therefore, modulating processes that are most likely mediated by M1, such as accuracy and speed, can have a significant impact at the level of a complex behavior. These findings concur with previous research studies that have found significant effects on finger-sequencing performance due to modulating neural excitability in M1 [11,36]. Notably, the finger-sequencing task involves a life-relevant behavior – typing – which would be an ideal target for motor-recovery therapies.

The results of the present study may be relevant to clinical research on motor recovery after stroke. Researchers have utilized non-invasive brain stimulation (tDCS and Transcranial Magnetic Stimulation) to promote motor recovery for stroke victims by either modulating excitability in the motor cortex of the damaged or the undamaged hemisphere [5,13-15,20,37-39]. Our results point to the possibility that stimulating both hemispheres simultaneously, with cathodal tDCS over the unaffected hemisphere and anodal tDCS over the affected hemisphere, may be an ideal montage for catalyzing motor recovery. Granted, the relevance of translational research with healthy subjects to treatments for stroke patients may be limited, due to differences in the mean age of study participants and stroke victims, and differences in brain function for the healthy and the damaged brain. However, this paper opens the way for future clinical research along these lines.

A methodological issue with dual-hemisphere tDCS deserves some attention. The tDCS technique involves the application of two kinds of stimulation – anodal and cathodal. Because of this, excitability always increases in one area of the brain (underneath the anode) and decreases in another area (underneath the cathode). This unique attribute of tDCS may be seen as a disadvantage if the aim of a study is to determine the effect of modulating excitability in only one part of the brain. Researchers have made attempts to work around this in two ways: 1) by placing an "active" electrode over the area of the brain that is the focus of the study, and a "reference" electrode over a different area that may be assumed to have no influence on the measured behavior [11]; 2) by increasing the size of the reference electrode in order to reduce its current density, with an aim to decrease the effect of the reference electrode on neural excitability [40]. However, some applications of tDCS utilize the potential for the technique to simultaneously apply anodal and cathodal stimulation. For example, tDCS has been employed to treat depression by simultaneously increasing excitability in the left pre-frontal cortex with anodal stimulation and decreasing excitability in the right pre-frontal cortex with cathodal stimulation, which may be ideal for stabilizing mood [41]. Similarly, the results of our study suggest that dual-hemisphere tDCS over the motor cortices may improve motor performance in the non-dominant hand more than uni-hemisphere tDCS, with just anodal stimulation over the non-dominant motor cortex.

## Conclusion

In conclusion, we found that dual-hemisphere stimulation (with a combination of cathodal tDCS over the dominant motor cortex and anodal tDCS over the non-dominant motor cortex) led to significantly greater improvements in finger-sequence performance for the non-dominant hand, compared to uni-hemisphere (with anodal tDCS over the non-dominant motor cortex) and to sham stimulation. These results are relevant to motor skill learning and to experimental treatment strategies for facilitating, and potentially enhancing, motor skills in patients with motor impairments.

## Authors' contributions

BWV designed and coordinated the study, carried out data collection and statistical analysis, and drafted the manuscript. CC carried out data collection, contributed to statistical analysis, and helped to revise the manuscript. GS participated in the design of the study and statistical analysis, and was involved in drafting and revising the manuscript. All authors read and approved the final manuscript.
